# Upper Extremity Function Assessment Using a Glove Orthosis and Virtual Reality System

**DOI:** 10.1177/1539449219829862

**Published:** 2019-03-02

**Authors:** Richard J. Adams, Allison L. Ellington, Kate Armstead, Kristen Sheffield, James T. Patrie, Paul T. Diamond

**Affiliations:** 1 Barron Associates Inc., Charlottesville, VA, USA; 2 Mary Baldwin University, Staunton, VA, USA; 3 UVA Encompass Health, Charlottesville, VA, USA; 4 The University of Virginia, Charlottesville, USA

**Keywords:** virtual reality, activities of daily living, instrumental activities of daily living, rehabilitation, occupational therapy

## Abstract

Hand motor control deficits following stroke can diminish the ability of patients to participate in daily activities. This study investigated the criterion validity of upper extremity (UE) performance measures automatically derived from sensor data during manual practice of simulated instrumental activities of daily living (IADLs) within a virtual environment. A commercial glove orthosis was specially instrumented with motion tracking sensors to enable patients to interact, through functional UE movements, with a computer-generated virtual world using the SaeboVR software system. Fifteen stroke patients completed four virtual IADL practice sessions, as well as a battery of gold-standard assessments of UE motor and hand function. Statistical analysis using the nonparametric Spearman rank correlation reveals high and significant correlation between virtual world-derived measures and the gold-standard assessments. The results provide evidence that performance measures generated during manual interactions with a virtual environment can provide a valid indicator of UE motor status.

## Introduction

Virtual world-based games, when combined with human motion sensing, can enable a neurorehabilitation patient to engage in realistic occupations that involve repetitive practice of functional tasks (Adams et al., 2018). An important component of such a system is the ability to automatically track patient movements and use those data to produce indices related to movement quality (Adams et al., 2015). Before these technology-derived measures can be considered relevant to clinical outcomes, criterion validity must be established. If validated, measures of virtual task performance may reasonably be interpreted as reflective of real-world functional status.

The objective of the study described in this article was to investigate the criterion validity of upper extremity (UE) performance measures automatically derived from sensor data collected during practice of simulated instrumental activities of daily living (IADLs) in a virtual environment. A commercially available SaeboGlove orthosis (SaeboGlove, 2018) was specially instrumented to enable tracking of finger and thumb movements. This instrumented glove was employed with an enhanced version of the Kinect sensor-based SaeboVR software system (SaeboVR, 2018) to enable employment of the hand, elbow, and shoulder in functional interactions with a virtual world. Performance measures were automatically generated during patient use through a combination of arm tracking data from the Kinect and the glove’s finger and thumb sensors. The primary investigational objective was to determine whether performance indices produced by this system for practice of virtual IADLs are valid indicators of a stroke patient’s UE motor status.

Previous investigations into combining hand tracking with video games to animate UE therapy have produced evidence for the efficacy of such interventions. A recent study compared a 15-session hand therapy intervention using a smart glove system and video games with a usual care regimen (Jung et al., 2017). Stroke patients using the smart glove system realized greater gains in Wolf Motor Function Test (WMFT) score compared with dosage-balanced conventional therapy. Another study investigating a similar glove-based device found significantly greater improvements in Fugl-Meyer and Box and Blocks test results for stroke patients who performed 15 sessions that included the technology-aided therapy compared with subjects receiving traditional therapy only (Carmeli, Peleg, Bartur, Elbo, & Vatine, 2011). An instrumented glove has also been used to support video game therapy that incorporates gripping-like movements and thumb-finger opposition (Chan et al., 2014).

Past research into the use of human motion tracking (sometimes referred to as motion capture) technologies for assessment of UE function has produced encouraging results. One group of researchers compared naturalistic point-to-point reaching movements with standardized reaching movements embedded in a virtual reality system, and established concurrent validity between the two (Schaefer & Hengge, 2016). An investigation involving a device that incorporates handgrip strength and pinch force measurement into virtual reality exercises provided support for system use as an objective evaluation of hand function, and for the potential of replacing conventional goniometry and dynamometry (Nica, Brailescu, & Scarlet, 2013). In another study, researchers employed a Kinect sensor in a software system that attempts to emulate a subset of the Fugl-Meyer Upper Extremity (FMUE) assessment (Kim, Cho, Baek, Bang, & Paik, 2016). Pearson correlation analysis between the Kinect-derived scores and traditionally administered FMUE test results for 41 hemiparetic stroke patients revealed a high correlation. Previous research involving the SaeboVR system established a moderate and statistically significant correlation between virtual IADL performance scores and the WMFT (Adams et al., 2015). Due to limitations of the Kinect optical tracking system, this previous work involving the SaeboVR system did not include tracking of grasp-release manual interactions with virtual objects (Adams et al., 2018). The present research addresses this limitation by fusing data from the Kinect sensor with data from finger- and wrist-mounted sensors on the SaeboGlove orthosis to reconstruct the kinematic pose of the patient’s UE.

The use of an assistive glove orthosis in the present work fills an important clinical need. Inability to bring the hand and wrist into a neutral position due to weakness and/or lack of finger extension can prevent participation in occupation-oriented functional practice (Lang, DeJong, & Beebe, 2009). A common technique to enable stroke patients to achieve a functional hand position (and thus participate in rehabilitation) is a dynamic splint that supports finger and/or wrist extension. When larger forces are necessary (e.g., to overcome abnormal muscle tone), an outrigger-type splint may be employed. For patients with no more than mild hypertonicity, a lower-profile device such as the SaeboGlove orthosis (SaeboGlove, 2018) can be used. Employment of an assistive glove orthosis in the context of virtual IADLs practice thus addresses some of the leading causes of hand motor control deficits following stroke and their adverse impact on ability to participate in daily activities (Kamper, Fischer, Cruz, & Rymer, 2006; Ng, Tsang, Kwong, Tse, & Wong, 2011).

## Method

### Participants

Candidates were recruited from a population of stroke patients receiving in-patient rehabilitation care, outpatient rehabilitation, or who had been previously discharged from rehabilitative care at the UVA Encompass Health Rehabilitation Hospital (Charlottesville, VA, USA). Table 1 includes the study characteristics. Of 17 patients enrolled in the study, 15 completed the protocol. One subject dropped out due to unrelated illness. A second subject was disenrolled due to an inability to adequately express an understanding of consent during re-verification at the beginning of the first post-consent study session.

**Table 1.** Patient Characteristics (*n* = 17).

Age, years, median (range)	67 (25-83)
Time since stroke onset in months, median (range)	12 (1-72)
Sex, M/F, *n* (%)	10 (59)/7 (41)
Race category, Black/White, *n* (%)	3 (18)/14 (82)
Ethnic category, Hispanic/non-Hispanic, *n* (%)	0 (0)/17 (100)
Side of hemiplegia, L/R, *n* (%)	10 (59)/7 (41)
Affected side dominance, dominant/nondominant, *n* (%)	9 (53)/8 (47)

All study activities were conducted under the auspices of the University of Virginia Institutional Review Board for Health Sciences Research (IRB-HSR). All study sessions took place in a private room within the UVA Encompass Health outpatient clinic between October 20, 2017, and February 9, 2018. Licensed Occupational Therapists trained in study procedures and registered with the IRB-HSR were responsible for all patient contact, recruitment, consent, and protocol administration.

Verification of inclusion/exclusion criteria was through a three-step process including an initial medical record review prior to recruitment, verbal confirmation prior to administration of consent, and an evaluation screen conducted by a study therapist following consent. Inclusion criteria included history of stroke with hemiplegia, ongoing stroke-related hand impairment, sufficient active finger flexion at the metacarpal phalangeal joint in at least one finger to be detected by visual observation by a study therapist, antigravity strength at the elbow to at least 45° of active flexion, antigravity shoulder strength to at least 30° each in active flexion and abduction/adduction, and 15° in active shoulder rotation from an upright seated position. Participants had visual acuity with corrective lenses of 20/50 or better and were able to understand and follow verbal directions. The study excluded patients with visual field deficit in either eye that would impair ability to view the computer monitor and/or with hemispatial neglect that would impair an individual’s ability to process and perceive visual stimuli. The study also excluded individuals with motor limb apraxia, significant muscle spasticity, or contractures of the muscles, joints, tendons, ligaments, or skin that would restrict normal UE movement.

### Materials

**Figure 1.**
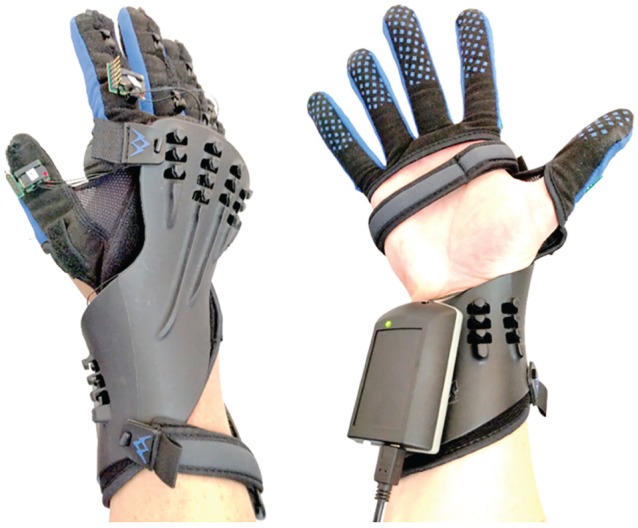
SaeboGlove orthosis with sensors to track grasp interactions.

A commercial SaeboGlove orthosis was fitted with wrist and finger motion sensors to permit tracking of finger joint angles during grasp-release interactions with a virtual environment. The instrumented glove orthosis is shown in Figure 1. The sensors were attached to the existing tensioner band hooks on the dorsal side of each glove finger. An electronics enclosure mounted to the palmar side of the SaeboGlove’s plastic wrist splint processes the sensor data and transmits information to a personal computer (PC) that hosts the modified SaeboVR software. Data from both the SaeboGlove-integrated sensors and from a Kinect sensor were used by a custom motion capture algorithm, which employs a human UE kinematics model to produce real-time estimates of arm, wrist, and finger joint angles.

The algorithms used for tracking of the shoulder and elbow have previously been described (Adams et al., 2015). Those algorithms were extended in the present work to encompass a hand articulation model with 27 degrees of freedom. The resulting motion capture solution includes joint angles, angular rates, joint positions, and velocity for the fingers, wrist, elbow, and shoulder. Using the tracking solution, PC software reconstructs the patient’s UE kinematic pose in real time. These data are used both to establish the kinematic pose of the patient’s avatar within the three-dimensional (3-D) virtual world and to generate motor performance indices.

**Figure 2.**
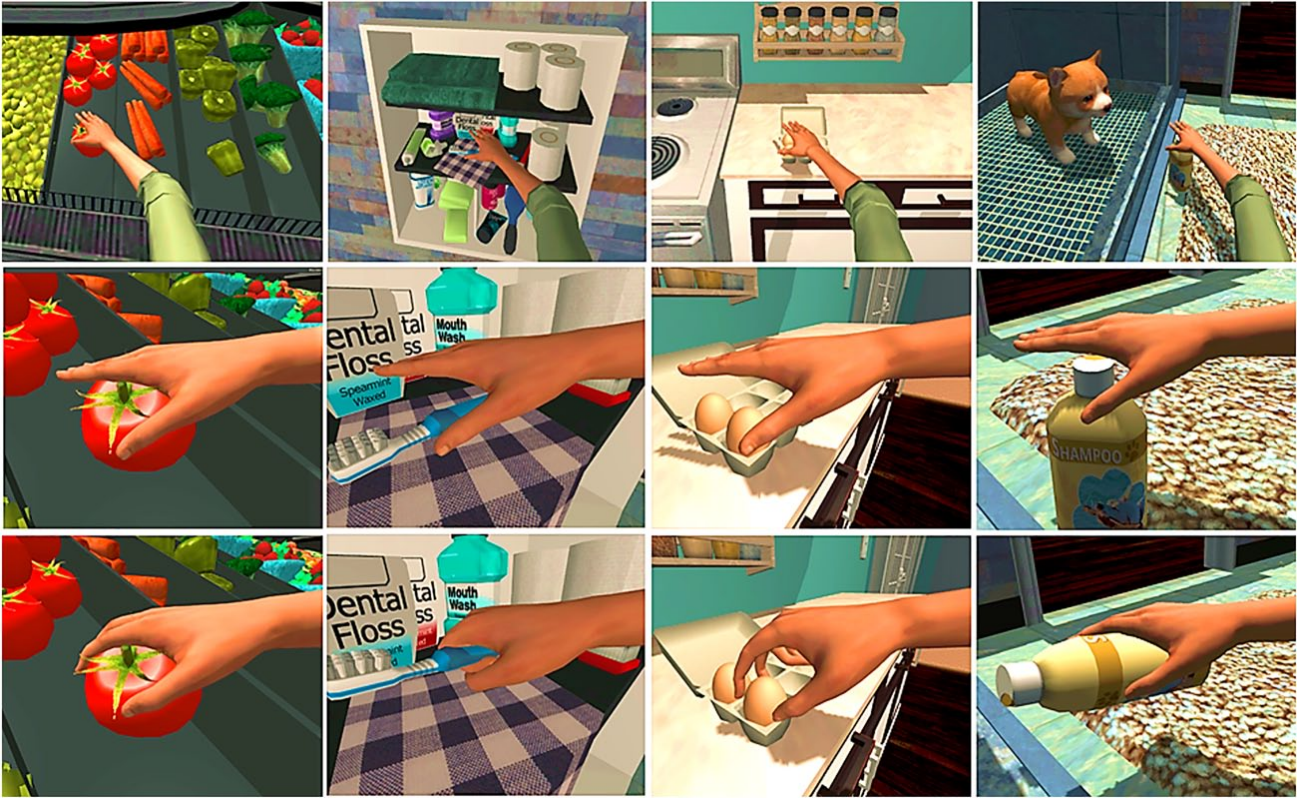
Virtual Instrumental Activities of Daily Living (IADLs) tasks involving reaching for and grasping an object.

The addition of the specially instrumented SaeboGlove extended the existing capabilities of the SaeboVR system to include manual grasp-release interactions with virtual objects. Existing SaeboVR activities include grocery shopping, putting away groceries, preparing breakfast, pet shopping, pet feeding, pet bathing, garden planting, garden harvesting, preparing dinner, organizing closet, volunteering at a soup kitchen, and a Balls and Boxes practice exercise. The IADLs are described in more detail in Adams et al. (2018). The activities involve task sequences that incorporate interactions with (picking up, placing, or manipulating) objects such as grocery items, pet care items, self-care instruments, utensils, clothing, drawers, cabinets, and appliances. Tasks also include sustained, stabilized contractions. For example, in pet bathing, the patient holds up and points a spray nozzle to wash bubbles off a puppy. Furthermore, the activities are interconnected in logical and realistic progressions. For example, in one sequence of three activities, a client first completes grocery shopping, then puts those grocery items away in the home, and finally prepares a meal with the purchased items. The existing Kinect-based SaeboVR system involves only the shoulder and elbow, with manual interactions automatically triggered by the placement of the patient avatar’s virtual hand over an object. In the glove-enhanced system reported on here, manual interaction with virtual objects requires both hand placement and a prescribed amount of flexion (for grasp) or extension (for release) in the involved digits. The amount of flexion and extension needed to trigger these grasp-release interactions is set through a simple calibration exercise, thereby allowing patients with only minimal active range of motion to successfully participate in the virtual occupations within the limits of his or her capabilities. Within the activities, each task is decomposed into subtasks (e.g., reaching for an item on a shelf) that incorporate point-to-point movements, with arrested motion (near-zero speed in Cartesian space) at well-defined start and stop locations in the virtual workspace. This task decomposition enables kinematic tracking data to be automatically parsed into data segments, which correspond to functional movements and can be individually analyzed for speed and quality of movement. Figure 2 shows typical interactions with the virtual environment involving reaching for and grasping objects. The patient sees the virtual world, including the arm and hand on the affected side, from a first-person perspective as he or she interacts with the environment. In addition to this visual feedback, auditory cues are presented for key interactions (e.g., picking up or placing an item). The system described here did not include haptic (vibratory or force) feedback.

### Procedures

Following an initial visit dedicated to consent, screening, glove fitting, and scheduling, participants were asked to return for four additional study visits. In each of these four study visits, patients used their stroke-affected arm to practice virtual IADLs using the specially instrumented SaeboGlove and the modified SaeboVR software system. In these four sessions, patients performed the garden harvesting, organizing closet, and Balls and Boxes activities, along with (time permitting) a choice of one or more of the other SaeboVR IADLs. In the final visit, following completion of the fourth virtual IADL session, a trained study therapist administered a battery of gold-standard tests of UE motor performance.

### Measures

Using the sensor-derived UE kinematic histories, the enhanced SaeboVR software system automatically generates functional metrics from the kinematic tracking data history acquired during patient interactions with the virtual world. The VR-produced indices are as follows:

- (Primary) *Subtask completion time* (*VR-SCT*): Representing speed of motion; time-to-complete is calculated for each functional movement involving manual interactions with a virtual object; the reported VR-SCT is the median time-to-complete over all functional movements (subtasks) performed by a patient in a session. The subtasks used in calculation of the kinematic measures involve point-to-point free space reaching movements. Although they are roughly equivalent in motor challenge level for a person with normal UE function, some patients may find some subtasks more challenging than others with respect to motor and/or cognitive demand. To reduce the potential for outliers to skew metrics calculated across subtasks, the VR indices are based on median values.
- (Secondary) *Normalized speed* (*VR-NS*): Representing smoothness of motion; for the extracted kinematic data segment for each functional movement, mean hand speed is divided by peak speed; the reported VR-NS is the median value of this quotient over all functional movements (kinematic data segments) performed by a patient in a session. The VR-NS metric has previously been found to be better correlated to traditional measures of motor performance than alternate smoothness measures (Adams et al., 2015).
- (Secondary) *Balls and Boxes* (*VR-BAB*) game score: A timed virtual world activity in which the patient earns points by picking up virtual balls and putting them in boxes; inspired by the traditional Box and Blocks test (Cromwell, 1976); the reported VR-BAB is the sum of points earned in 1 min.

It has previously been shown that learning-associated variability in patient performance using the SaeboVR system is significantly reduced by the third session (Adams et al., 2015). After the third use, virtual IADL metrics generated from individual functional movements can be considered reflective of motor performance versus cognitive status. Therefore, only the VR indices generated in the fourth and final session of this study were used in the subsequent correlation analysis. In this final study session, a study therapist also administered a battery of gold-standard tests of UE motor performance. These included the following:

- (Primary) WMFT (Morris, Uswatte, Crago, Cook, & Taub, 2001; Wolf et al., 2001) was chosen as the primary measure for investigation of validity as it involves timed completion of integrative functional movements, and thus is the most ADL-oriented of available assessments. The test has been shown to have high interrater reliability, validity, and internal consistency (Wolf et al., 2001). The WMFT includes quantitative and qualitative scales. WMFT-TIME is a quantitative measure calculated using the average time to complete the tasks. The WMFT Functional Assessment (FA) score is a qualitative assessment based on scoring of each functional task using a 6-point ordinal rating scale that ranges from 0 (*no use of the affected hand attempted*) to 5 (*normal function*). A total WMFT-FA is calculated by taking the average across all 15 UE tasks.
- (Secondary) *FMUE* (Fugl-Meyer, Jaasko, Leyman, Olsson, & Steglind, 1975) is one of the most widely used and accepted quantitative measures of motor function in stroke patients. It is used in both clinical and research settings. Individual patient movements associated with specific motor functions are scored using a 3-point ordinal scale (0, 1, or 2). Higher scores correspond to higher levels of motor function.
- (Secondary) *Box and Blocks Test* (Cromwell, 1976) is a well-accepted measure of manual dexterity that has been shown to have high test–retest reliability and validity as a predictor of UE status and function (Desrosiers, Bravo, Hébert, Dutil, & Mercier, 1994).
- (Secondary) *Motor activity log* (MAL; Uswatte, Taub, Morris, Vignolo, & McCulloch, 2005; van der Lee, Beckerman, Knol, de Vet, & Bouter, 2004) is a structured interview assessing Quality of Movement and Amount of Use for real-world use of the affected arm in 30 ADLs. For each of the ADLs, the subject provides a self-assessment of both the frequency that the activity is performed (MAL-Amount) and the quality (MAL-HowWell).

Study therapists were practiced in use of the instruments both from previous studies and from use in clinical practice. Prior to being certified to administer the protocol, the therapists received additional training and demonstrated proficiency in consistent application of the rubrics used in each assessment. The therapist administering the gold-standard measures was effectively blinded to the collection of software-generated outcome measures, as they had no fore-knowledge of how those measures were generated by the software or ability to impact how they were derived using the computerized motion tracking system.

### Data Analysis

Consistent with the IRB-approved protocol, the primary relationship under consideration was the correlation between the software-generated VR-SCT measure and the WMFT-TIME gold-standard clinical measure. Pre-study power analysis indicated that if at least 15 participants completed the study, and if the true bivariate correlation between these measures was at least .62, there would be 80% statistical power to reject the null hypothesis that the true correlation is zero. The level of correlation between each of the secondary VR measures and each of the gold-standard clinical measures was also considered to support investigation of criterion validity and identification of the most promising computer-generated metrics for future exploration.

To determine criterion validity, bivariate relationships between VR software-derived measures and the gold-standard clinical measures were estimated by way of the nonparametric Spearman rank correlation (*r_s_*). The primary outcome measures for determining criterion validity were VR-SCT and WMFT-TIME. Analysis for secondary outcomes included the bivariate relationship between each VR measure and each gold-standard measure. For each correlation analysis, a 95% confidence interval (CI) is formed. A *p* ≤ .05, two-tailed decision rule was used for rejecting the null hypothesis. Correlation coefficients ≥ .7 were considered to reflect high criterion validity (Hinkle, Wiersma, & Jurs, 2003). Coefficients in the range .5 ≤ *r_s_* < .7 corresponded to moderate criterion validity. Coefficients between .3 ≤ *r_s_* < .5 were considered low, with lesser values deemed negligible.

The collection of data using four different clinical tests of UE motor performance (two with subscales) in this study also presented an opportunity to analyze the bivariate relationships between these gold-standard assessments and thus contribute to the body of knowledge regarding their concurrent validity. Accordingly, analysis for secondary outcomes presented here includes generation of Spearman correlations and CIs for the each of the 15 possible combinations of these clinical measures.

## Results

### Primary Outcome

**Figure 3.**
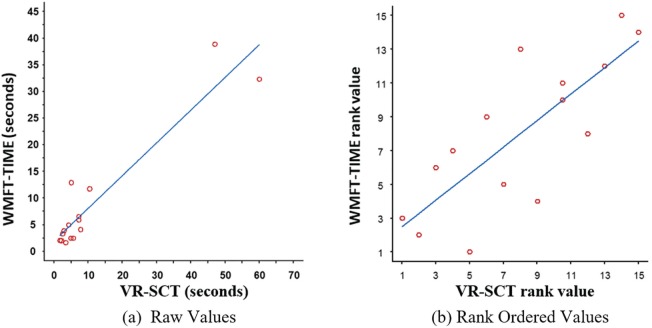
Bivariate relationships between VR-SCT and WMFT-TIME. *Note.* SCT = subtask completion time; WMFT = Wolf Motor Function Test.

Figure 3 shows the bivariate relationship between the primary VR outcome measure, VR-SCT, and the primary gold-standard clinical measure, WMFT-TIME. The plot on the left shows the relationship between the raw values of these measures. The plot on the right shows the corresponding rank-order values used in the nonparametric Spearman correlation analysis. The ordinary least squares regression line of best fit is shown in blue. Red circles show the outcome data for each subject (*N* = 15). The first row of Table 2 (in bold) summarizes the primary test for criterion validity. The Spearman correlation between VR-SCT and WMFT-TIME is *r_s_* = .78 (95% CI = [0.44 0.93], *p* = .001), revealing a high and significant correlation between the software-derived measure and the clinical assessment of hand function. The criterion validity of the primary VR outcome measure is thus confirmed.

**Table 2.** Statistical Assessment of the Relationship Between VR-SCT and Gold-Standard Measures.

*X*	*Y*	Spearman *r_s_*	95% CI		*p* value
**VR-SCT**	**WMFT-TIME**	**.78**	**0.44**	**0.93**	**.001**
VR-SCT	WMFT-FA	−.66	−0.88	−0.20	.008
VR-SCT	Box and Blocks	−.81	−0.94	−0.50	<.001
VR-SCT	FMUE	−.74	−0.91	−0.35	.002
VR-SCT	MAL-Amount	−.47	−0.80	0.07	.077
VR-SCT	MAL-HowWell	−.56	−0.84	−0.06	.029

*Note.* SCT = subtask completion time; CI = confidence interval; WMFT = Wolf Motor Function Test; FA = functional assessment; FMUE = Fugl-Meyer upper extremity; MAL = motor activity log.

### Secondary Outcomes

Table 2 also summarizes the results of analysis of the bivariate relationships between the primary software-generated metric, VR-SCT, and each additional gold-standard measure that was considered in the study. VR-SCT has high correlation to both Box and Blocks and FMUE. Correlations to the WMFT-FA and the MAL-HowWell subscales are moderate, but significant. The relationship to the MAL-Amount score is low and fails the test for significance.

Table 3 summarizes the analysis of the correlation between the VR-NS (normalized speed, related to smoothness of movement) and the gold-standard clinical measures. We see that the software-generated smoothness metric has low correlation to WMFT-TIME, Box and Blocks, and FMUE, and negligible correlation to WMFT-FA and to the MAL subscales. The relationships between the VR-BAB and the gold-standard measures are summarized in Table 4. The software-derived VR-BAB score is highly correlated to all of the clinical measures, with the exception of the MAL subscales.

**Table 3.** Statistical Assessment of the Relationship Between VR-NS and Gold-Standard Measures.

*X*	*Y*	Spearman *r_s_*	95% CI		*p* value
VR-NS	WFMT-TIME	−.40	−0.77	0.15	.135
VR-NS	WMFT-FA	.22	−0.35	0.67	.436
VR-NS	Box and Blocks	.39	−0.17	0.76	.148
VR-NS	FMUE	.41	−0.15	0.77	.133
VR-NS	MAL-Amount	.29	−0.28	0.71	.297
VR-NS	MAL-HowWell	.26	−0.31	0.69	.357

*Note.* NS = normalized speed; CI = confidence interval; WMFT = Wolf Motor Function Test; FA = functional assessment; FMUE = Fugl-Meyer upper extremity; MAL = motor activity log.

**Table 4.** Statistical Assessment of the Relationship Between VR-BAB and Gold-Standard Measures.

*X*	*Y*	Spearman *r_s_*	95% CI		*p* value
VR-BAB	WFMT-TIME	−.77	−0.92	−0.40	.001
VR-BAB	WMFT-FA	.79	0.46	0.93	<.001
VR-BAB	Box and Blocks	.77	0.42	0.92	.001
VR-BAB	FMUE	.80	0.47	0.93	<.001
VR-BAB	MAL-Amount	.33	−0.23	0.73	.229
VR-BAB	MAL-HowWell	.50	−0.04	0.81	.060

*Note.* BAB = balls and boxes; CI = confidence interval; WMFT = Wolf Motor Function Test; FA = functional assessment; FMUE = Fugl-Meyer upper extremity; MAL = motor activity log.

To assess the concurrent validity of the gold-standard instruments administered in this study, we also analyzed the bivariate correlations between each of these measures. Table 5 shows a very high correlation between both WMFT subscales and the Box and Blocks test. All the remaining bivariate correlations are high, with the exceptions of those involving the MAL subscales.

**Table 5.** Statistical Assessment of the Relationship Between Gold-Standard Measures.

*X*	*Y*	Spearman *r_s_*	95% CI		*p* value
WMFT-TIME	WMFT-FA	−.89	−0.96	−0.69	<.001
WMFT-TIME	Box and Blocks	−.99	−1.00	−0.95	<.001
WMFT-TIME	FMUE	−.84	−0.95	−0.55	<.001
WMFT-TIME	MAL-Amount	−.55	−0.83	−0.03	.035
WMFT-TIME	MAL-HowWell	−.78	−0.92	−0.43	.001
WMFT-FA	Box and Blocks	.90	0.72	0.97	<.001
WMFT-FA	FMUE	.85	0.60	0.95	<.001
WMFT-FA	MAL-Amount	.52	0.00	0.82	.045
WMFT-FA	MAL-HowWell	.68	0.24	0.89	.005
Box and Blocks	FMUE	.86	0.61	0.95	<.001
Box and Blocks	MAL-Amount	.59	0.10	0.85	.020
Box and Blocks	MAL-HowWell	.81	0.50	0.94	<.001
FMUE	MAL-Amount	.62	0.14	0.86	.014
FMUE	MAL-HowWell	.75	0.38	0.92	.001
MAL-Amount	MAL-HowWell	.87	0.63	0.96	<.001

*Note.* CI = confidence interval; WMFT = Wolf Motor Function Test; FA = functional assessment; FMUE = Fugl-Meyer upper extremity; MAL = motor activity log.

## Discussion

This work provides strong evidence that performance measures generated by software using motion capture data acquired during a stroke patient’s manual interactions with a virtual environment can provide a valid indicator of UE motor status. The bivariate relationship between the primary outcome measures, VR-SCT and the WMFT-TIME, shows a high correlation that is similar in magnitude to those observed between well-accepted gold-standard measures (e.g., between WMFT-TIME and FMUE). It may therefore be possible to employ a software system such as SaeboVR, which provides virtual practice of IADLs, to systematically monitor and document a patient’s progress during a course of therapy. The computerized assessment has the advantages that it occurs within an intervention and does not require special training to administer or score. As the system continually gathers and reports data about a patient’s motor performance during therapy, this VR system constitutes a valid reassessment each time it is used. In a tele-therapy context, it may be possible to monitor a patient’s motor status during independent practice sessions.

Although software-generated UE motor performance measures have been studied previously (Adams et al., 2015; Kim et al., 2016), this work represents the first time a glove orthosis has been endowed with sensors to track manual (grasp-release) interactions with objects in virtual IADL practice. The inclusion of wrist and finger joint angle tracking resulted in a marked increase in the correlation of software-generated VR-SCT with the gold-standard WMFT-TIME when compared with Kinect-only tracking of the shoulder and elbow. In a previous study that did not include tracking of grasp-release interactions, the Spearman correlation between these variables was found to be moderate, *r_s_* = .56 (Adams et al., 2015). With the introduction of the specially instrumented SaeboGlove in this study, the bivariate relationship increases to a much higher *r_s_* = .78. The use of the instrumented glove orthosis for wrist and finger tracking, in conjunction with an optical sensor to track the arm, provides a higher quality reconstruction of UE kinematics and superior metrics for monitoring functional motor status.

The inclusion of VR-NS in this study was inspired by previous research at the Massachusetts Institute of Technology (MIT) on movement smoothness during stroke recovery (Rohrer et al., 2002). The metric is based on the presumption that, for a given functional movement, motion can be considered smooth to the extent that the mean speed of motion is close to the peak speed achieved. It is notable that the low correlation between the VR-NS metric and the gold-standard assessments reported in this article is consistent with MIT’s findings and with values reported in a related study (Adams et al., 2015). It thus appears that smoothness of motion may only be indirectly reflected in clinical measures, and that the value of smoothness-related measures to clinicians is yet to be established.

One of the limitations of this study is a modest sample size. Consequently, the significance of less-than-moderate correlations could not be established, and the CIs reported may be larger if a greater number of subjects had been involved. Another limitation is that inclusion criteria only permitted participation by patients with mild to moderate impairment, and excluded individuals with apraxia, spasticity, contractures, neglect, and/or inability to understand or follow verbal directions. The study findings therefore cannot be generalized to more severely impaired subjects without additional research.

Within the profession of occupational therapy, use of occupation in the therapeutic process is a central tenet. Existing research has found that both occupation-based and repetitive task intervention approaches are effective in promoting recovery following stroke (Skubik-Peplaski, Custer, Powell, Westgate, & Sawaki, 2017). In the present study, these two approaches are combined, with a patient performing repeated reaching and grasping movements within the context of virtual IADL participation. Therefore, these results establishing the validity of tracking hand movements within the virtual IADL practice context is notable. This study has the potential to impact the future practice of occupational therapy by aligning new technology with the foundational beliefs of the profession.

## Conclusion

This study provides evidence that indices produced by a sensor-based system for manual practice of virtual IADLs are valid indicators of a stroke patients’ UE motor performance. These computer-derived measures of virtual task performance can be considered representative of real-world functional status.
